# Comparing fatal crash risk factors by age and crash type by using machine learning techniques

**DOI:** 10.1371/journal.pone.0302171

**Published:** 2024-05-06

**Authors:** Abdulaziz H. Alshehri, Fayez Alanazi, Ahmed. M. Yosri, Muhammad Yasir

**Affiliations:** 1 Department of Civil Engineering, College of Engineering, Najran University, Najran, Saudi Arabia; 2 Department of Civil Engineering, College of Engineering, Jouf University, Sakaka, Saudi Arabia; 3 Department of Civil Engineering, Swedish College of Engineering and Technology, Wah Cantt, Pakistan; Sunway University, MALAYSIA

## Abstract

This study aims to use machine learning methods to examine the causative factors of significant crashes, focusing on accident type and driver’s age. In this study, a wide-ranging data set from Jeddah city is employed to look into various factors, such as whether the driver was male or female, where the vehicle was situated, the prevailing weather conditions, and the efficiency of four machine learning algorithms, specifically XGBoost, Catboost, LightGBM and RandomForest. The results show that the XGBoost Model (accuracy of 95.4%), the CatBoost model (94% accuracy), and the LightGBM model (94.9% accuracy) were superior to the random forest model with 89.1% accuracy. It is worth noting that the LightGBM had the highest accuracy of all models. This shows various subtle changes in models, illustrating the need for more analyses while assessing vehicle accidents. Machine learning is also a transforming tool in traffic safety analysis while providing vital guidelines for developing accurate traffic safety regulations.

## 1 Introduction

Traffic crashes are a significant public health concern worldwide, with over 1.5 million people losing their lives annually. This study introduces a novel approach by applying machine learning methods to analyze risk factors of crashes by age group and type of crash, providing new insights into traffic safety. In Saudi Arabia, road accidents are a leading cause of injury-related deaths, particularly in urban areas. While statistical models have traditionally been used to evaluate road accident severity, the advent of machine learning technologies offers a promising alternative. Machine learning techniques can understand complex, non-linear relationships between data elements, potentially improving accuracy and consistency in traffic safety analysis. However, there is a notable gap in research, especially in analyzing different driving hazards. This study aims to fill this gap by employing various supervised learning techniques to analyze a dataset of traffic incidents in the city, focusing on individual incidents and age categories. Advanced machine learning methods and comparative analysis set this study apart from previous research, with the potential to significantly impact traffic safety research and inform the creation of effective traffic safety regulations.

Several decades have passed since statistical models were used to assess the severity of road accidents and traffic safety. On the other hand, technologies based on machine learning can potentially deliver an alternative revolution. It is acknowledged that machine learning approaches may be used to comprehend intricate, non-linear interactions between various data components [1]. This has resulted in greater anticipated accuracy and consistency than traditional methods [2]. Research fields are falling behind, particularly regarding research on various driving dangers [3]. This is even though there have been numerous advancements in technological development. Such a large study gap highlights the need to do individualized research to identify the myriad of elements contributing to road accidents [4, 5]. Motor vehicle accidents may be caused by various factors, which is why this is the case. The role of age in intensifying vehicular accident fatalities has been proven after a detailed review of appropriate resources. Serious traffic accidents involving young drivers frequently occur, mainly due to the lack of experience on their part [6, 7]. However, senior drivers have a greater chance of severe wreck involvement. These are associated with aging issues, for example, slow response to stimuli and limited senses. Similarly, it should be mentioned that male drivers are typically involved in severe accidents [8, 9], thus underscoring the substantial impact of the phenomenon.

Moreover, the qualities directly related to the accidents contribute significantly to the magnitude of such accidents [10]. Among these factors are the speed at which the car was hit by the other vehicle, also known as the second automobile [12], the number of cars involved in the incident [13], and the congestion level. Other external factors contributing to the seriousness of accidents include driving at night and unfavorable weather conditions. Other significant factors influencing traffic accident results include the nature of the road where the accident occurred and the collision structure. However, this knowledge is restricted due to the absence of extensive research studies comparing these different communities [11].

The demographic trends impacting road accident incidence have been thoroughly documented in high-income countries such as the United States and Sweden, thanks to the research conducted [12]. Several pieces of empirical data demonstrate, time and time again, that certain demographic groups are disproportionately represented in accidents that result in catastrophic consequences [13–15]. It is important to note that this category includes senior drivers who are 65 or older and younger male drivers older than 25. It is also concerning because this demographic trend raises fundamental issues about the suitability and effectiveness of the rules for driver education and road safety [16, 17]. This is especially true concerning the complex needs of these vulnerable groups. Despite this, there is a disturbing lack of data and documentation in countries with lower and intermediate incomes, such as Saudi Arabia [18, 19]. This is especially alarming considering the distinct road conditions and regulatory regimes distinctive to these places. At the same time, the specific circumstances of the accident are pretty important when examining traffic safety. Compared to rear-end accidents, other collisions, such as head-on collisions, side-impact collisions, and wrecks involving a single vehicle, are often associated with more severe results [20, 21]. On the other hand, the complicated hazards associated with every type of accident and how these dangers vary depending on the drivers’ demographics have not been examined to a considerable extent. Given the importance of creating more effective and targeted safety measures and interventions, which need a full awareness of the relationship between accident type and driver characteristics, this absence of information is considerable [22, 23]. This is because the development of these measures and interventions is vital.

A progressive progression has characterized the application of machine learning to studying traffic safety throughout its history. Using complex approaches such as gradient boosting [24] and decision trees tuned to mitigate class imbalance [25], strict regression models have been shown to perform better than other models when it comes to forecasting the severity of failures [26]. One of the reasons why machine learning is powerful is that it can get into very complex forms of data in search of hidden information. Through this, various nuances, such as those ignored by the common-place analytical methods, are brought out. However, such elaborate analytical procedures are hardly used in any study. The lack of enough data restricts comprehensive assessments of various kinds of risk associated with drivers’ subsets at once in a single dataset set [27, 28].

In recent years, the proliferation of traffic-related fatalities has escalated into a global concern, underscoring the urgent need for comprehensive research in traffic safety. Despite considerable advancements in vehicle technology and road safety measures, a critical examination of existing literature reveals a notable gap: a scarcity of in-depth comparative analyses on the determinants of fatal crash risks, mainly differentiated by age groups and crash types, using sophisticated machine learning techniques. This oversight highlights a significant shortfall in our current understanding and approach to mitigating traffic fatalities. Therefore, this study is conceived to bridge this gap by employing advanced machine learning methodologies to dissect and compare the underlying risk factors contributing to fatal crashes across different age demographics and crash scenarios. By doing so, this research aims to provide a nuanced understanding of the complexities involved in traffic accidents, thereby offering targeted insights for developing more effective safety interventions. Such a contribution is pivotal for advancing traffic safety research and formulating policies that can significantly reduce the incidence of fatal traffic accidents.

## 2 Related work

The study of traffic crash severity and the application of machine learning techniques to enhance road safety have been subjects of considerable interest in recent research. Several studies have utilized various statistical and machine learning models to analyze factors influencing the severity of road accidents. For instance, Abdelwahab and Abdel-Aty (2001) employed logistic regression models to examine the impact of driver characteristics on crash severity, highlighting the significance of age, gender, and driving experience. Similarly, Chang and Chen (2005) used decision tree models to identify key factors contributing to severe injuries in traffic crashes, emphasizing the role of environmental and vehicle-related variables.

Recent advancements in machine learning have led to the exploration of more sophisticated techniques in traffic safety research. Kunt and Yasar (2017) applied neural networks to predict the severity of road accidents, demonstrating the potential of these models to capture complex relationships between variables. Additionally, Li et al. (2019) employed support vector machines to classify accident severity, showcasing the effectiveness of this method in handling imbalanced datasets.

Despite these advancements, there remains a gap in the literature concerning the comprehensive analysis of traffic crash risk factors across different age groups and incident types using machine learning methods. Most studies focus on specific aspects of traffic safety, such as driver behavior or environmental factors, without a holistic comparison of various risk factors. This study aims to bridge this gap by employing various supervised learning techniques to analyze a complete dataset of traffic incidents in Jeddah City. By comparing the risk factors associated with different age groups and types of incidents, this research seeks to provide a more nuanced understanding of traffic safety, contributing to the development of targeted intervention programs and customized safety measures.

## 3 Data

### 3.1 Data and methods

This study used crash data covering January 2020 to December 2022. The dataset includes various variables related to traffic crashes, such as crash type, location, time, and involved parties’ demographics. We employed machine learning algorithms to analyze the data and identify significant risk factors for different age groups and types of crashes.

### 3.2 Splitting data for training and testing

The study used a stratified split approach to ensure the representativeness of our training and testing sets. This method maintained the distribution of various crash types and age groups in both sets, allowing for a more accurate and reliable analysis. The training set was used to build the machine learning models, while the testing set was used to evaluate their performance and validate the findings.

### 3.3 Ethical considerations to take into account

The research is conducted following ethical norms, and it is made sure that the data does not include any information that may be used to identify individuals. To protect the anonymity of the victims, we focused primarily on aggregate accident facts. This methodology follows the ethical norms of our institute, and the study was carried out with complete ethical approval. Every step observed in obtaining and treating the data was carried out carefully, ensuring the data subjects’ privacy and confidentiality were protected.

### 3.4 Data source and description

A set of information on 877 traffic accidents in the city from 2019–2023, created by the Traffic Police Department. This comprises 29 different variables, as shown in Table 1. These datasets include information about vehicle identity, victims’ profiles, and the involved infrastructures, including weather conditions and the type and degree of each crash. To facilitate analysis, the severity of injuries has been categorized into two groups: Fatal (295 incidences) and non-fatal (582 incidents). The comprehensiveness of this dataset, as well as the usefulness of the data in terms of addressing the goals of the research, helped to justify the selection of this dataset. This dataset is especially useful for gaining an understanding of the trends and variables that contribute to traffic accidents in the setting of metropolitan Saudi Arabia.

**Table 1:** The city crash data feature and variable.

Feature	Description	Data Type	No. of Categories	Range
Crash_Type	Type of crash (e.g., Collision, Side-collision, etc.)	object	5	Collision, Overturning, Rollover, Run-off-road
Driver_Age	Age of the driver involved in the crash	int64	74	10–99
Driver_Gender	Gender of the driver (Male, Female, Other)	object	3	Male, Female, male
Location	Location of the crash	object	1	The city
Injury Severity	Severity of injuries (Fatal, Non-Fatal)	object	2	Non-Fatal, Fatal
Date	Date of the crash occurrence	object	257	[Various dates from 2019–2021]
Time	Time of the day when the crash occurred	object	156	[Various times throughout the day]
Weather_Condition	Weather conditions at the time of the crash	object	3	Sunny, Dust Storm, Rain
Day	The day of the week when the crash occurred	object	7	Monday, Wednesday, Sunday, Friday, Tuesday, Saturday, Thursday
Month	The month of the year when the crash occurred	object	12	May, March, April, December, January, July, June, February, November, October, August, September
Road_Name	Name of the road where the crash occurred	object	10	Falasteen Road, Heraa Street, Al Andalus Street,. . .
Road_Type	Type of road (e.g., City Street, Rural Road)	object	4	Desert Road, Rural Road, Expressway, City Street
Lanes	Number of lanes on the road	int64	3	2–4
Speed_Limit	The speed limit on the road where the crash occurred	int64	4	0–100
Infrastructure_Condition	Condition of the infrastructure (Good, Average, Poor)	object	3	Poor, Moderate, Good
Light_Condition	Light conditions during the crash (Day, Night, Dawn, etc.)	object	5	Day, Dawn, Night, Sunday, day
Driver_License_Status	Status of the driver’s license (Valid, Suspended)	object	2	Valid, Suspended
Traffic_Violations	Number of traffic violations recorded	int64	8	0–45
Vehicle_Speed	Speed of the vehicle involved in the crash	int64	30	30–155
Vehicle Type	Type of the vehicle involved (Sedan, 4x4, etc.)	object	8	SUV, Large SUV, Mini Bus, Delivery Truck, Luxury Car,. . .
Reason	Reason for the crash (e.g., Aggressive Driving, Over Speeding)	object	7	Distraction, Aggressive Driving, Faulty Vehicle.
Road_Conditions	Road conditions (Wet, Dry, Under Construction, etc.)	object	6	Winding Road, Obstacle Present, Poor Maintenance.
Number_of_Vehicles_Involved	Number of vehicles involved in the crash	int64	5	1–5
Traffic_Density	Traffic density at the time of the crash (Low, Medium, High)	object	3	Medium, Low, High
Time_of_Day	Time of day when the crash occurred (Morning, Afternoon, Evening, Night)	object	4	Morning, Afternoon, Evening, Night
Driver_Experience	Experience level of the driver (e.g., 1–5 years, 6–10 years)	object	4	6–10 years, 11–20 years, 1–5 years, 20+ years
Cause_of_Accident	Primary cause of the accident (e.g., Distraction, Weather Condition)	object	5	Overspeeding, Weather Conditions, Rule Violation.
Is_Fatal	Indicates if the crash was fatal (0 for No, 1 for Yes)	int64	2	0 (No), 1 (Yes)

While this study provides valuable insights into the risk factors associated with fatal crashes, it is essential to note that our dataset was explicitly limited to deadly crashes. Property damage crashes were not considered in our analysis. This exclusion was deliberate, as our primary objective was to compare the risk factors associated with severe outcomes. However, this limitation means that our findings may not directly apply to crashes resulting in property damage but no fatalities. Future research should consider including property damage crashes in their analysis to perform a more in-depth examination of the crash outcomes and provide a comprehensive understanding of the factors contributing to fatal and non-fatal crashes.

### 3.5 Rationale for exclusion of gender in analysis

In the initial design of our study, as outlined in Table 2, gender was acknowledged as a significant factor in previous research concerning crash risk factors. However, upon further deliberation, it was decided to exclude gender from the primary analysis for this specific investigation. This decision was not made lightly and warrants clarification.

Our research focuses predominantly on environmental and vehicular factors that influence crash risks. The decision to exclude gender from our analysis stems from a targeted approach that prioritizes these elements. The study aimed to isolate and examine the impact of these factors without the variability introduced by demographic variables. This approach aligns with our study’s objectives, intending to provide a concentrated analysis of areas less explored in existing literature.

It is crucial to acknowledge that excluding gender does not negate its importance in traffic safety research. Previous studies have demonstrated significant gender differences in crash involvement rates and risk-taking behaviors. Recognizing this, the study’s scope was designed to complement, not replace, the comprehensive body of work that includes gender as a critical variable.

### 3.6 Future research direction

We advocate for future research focusing on the gender effects on crash risk factors. Such studies are imperative to fully understand crash risks’ multifaceted nature and develop targeted interventions. Our research lays the groundwork for these investigations by highlighting the need for a diverse approach encompassing demographic and non-demographic factors in traffic safety analysis.

### 3.7 Data visualisation

**Fig 1:**
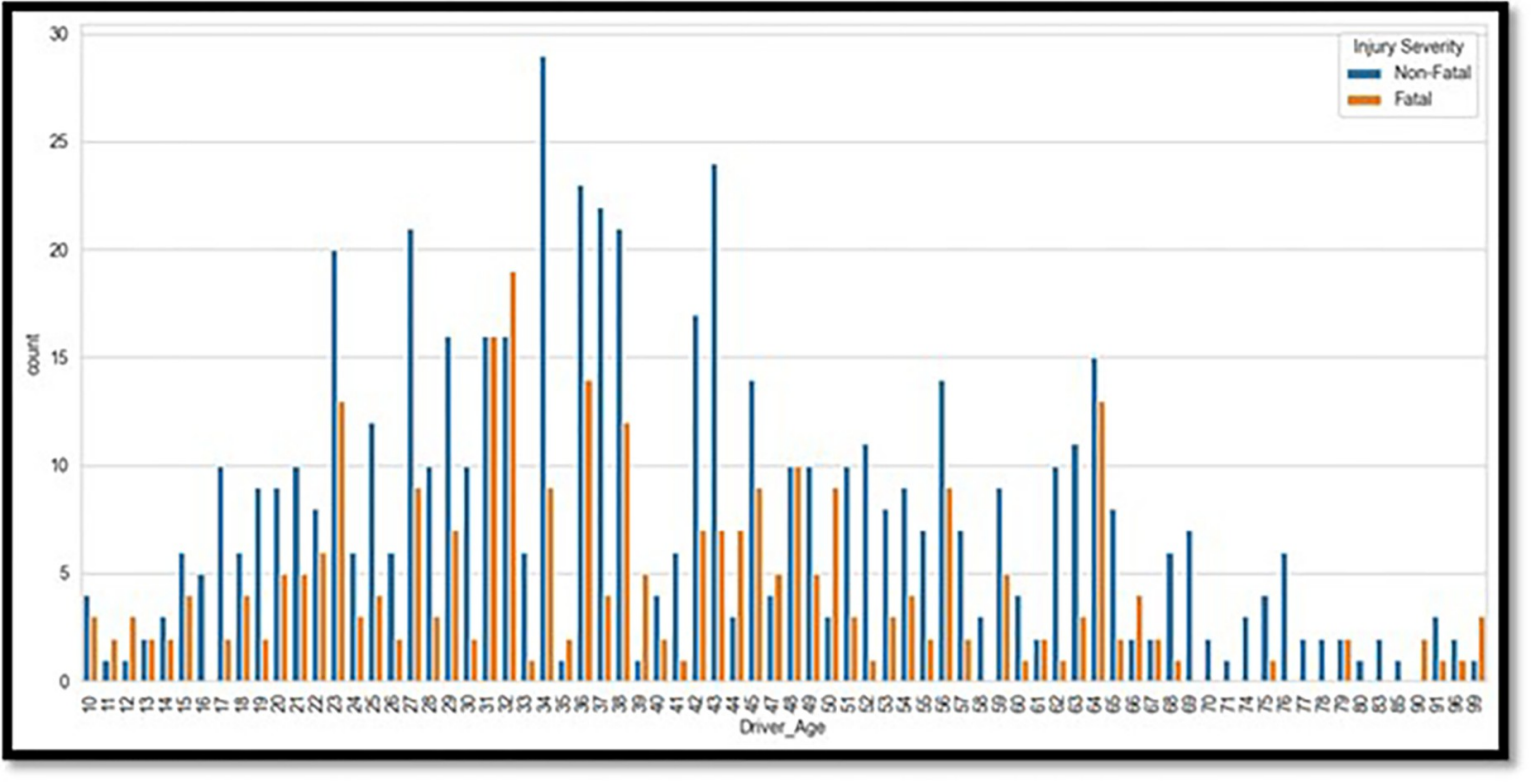
Distribution of driver age peaks in the 20–50-year range.

**Fig 2:**
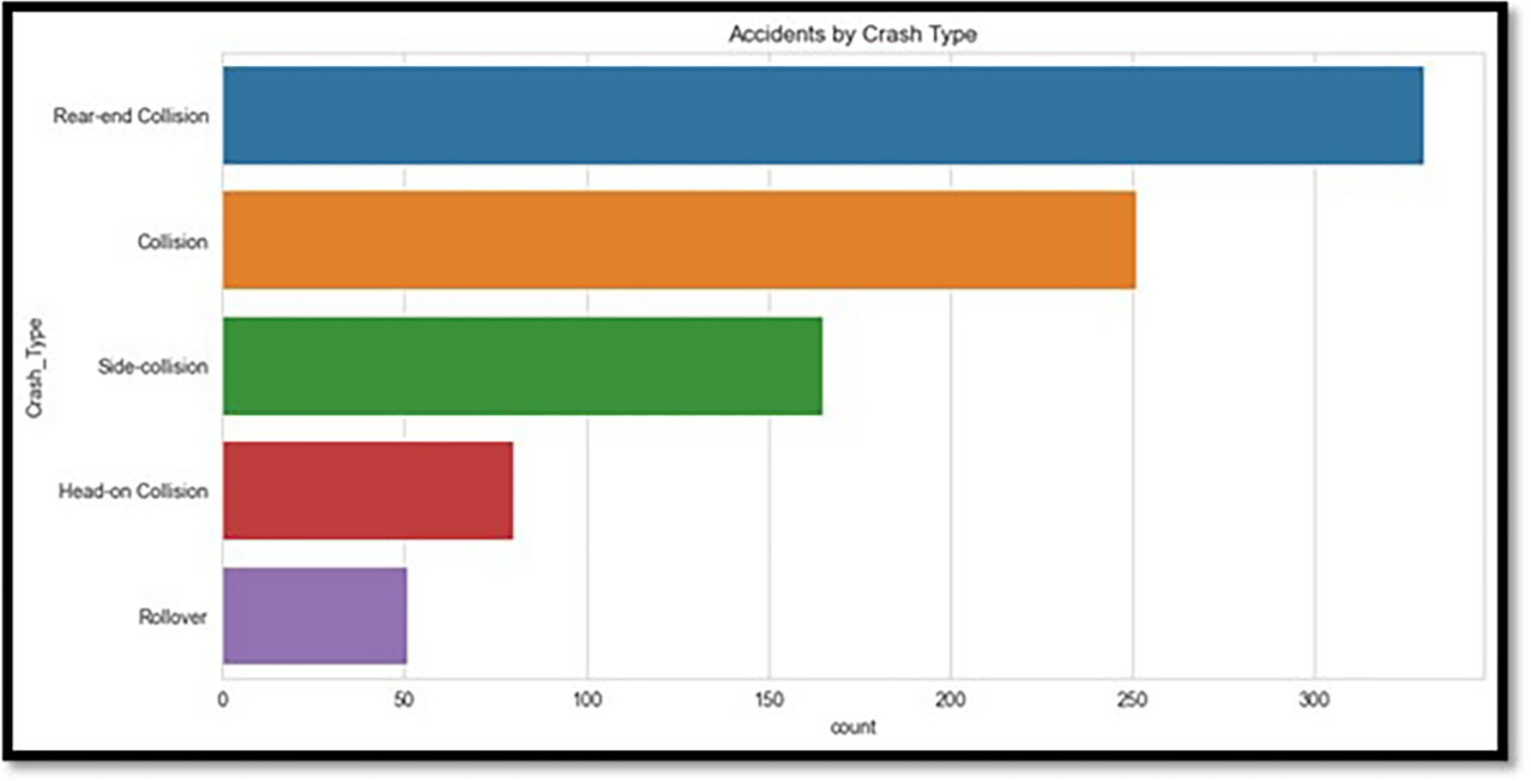
Accidents by crash type.

**Fig 3:**
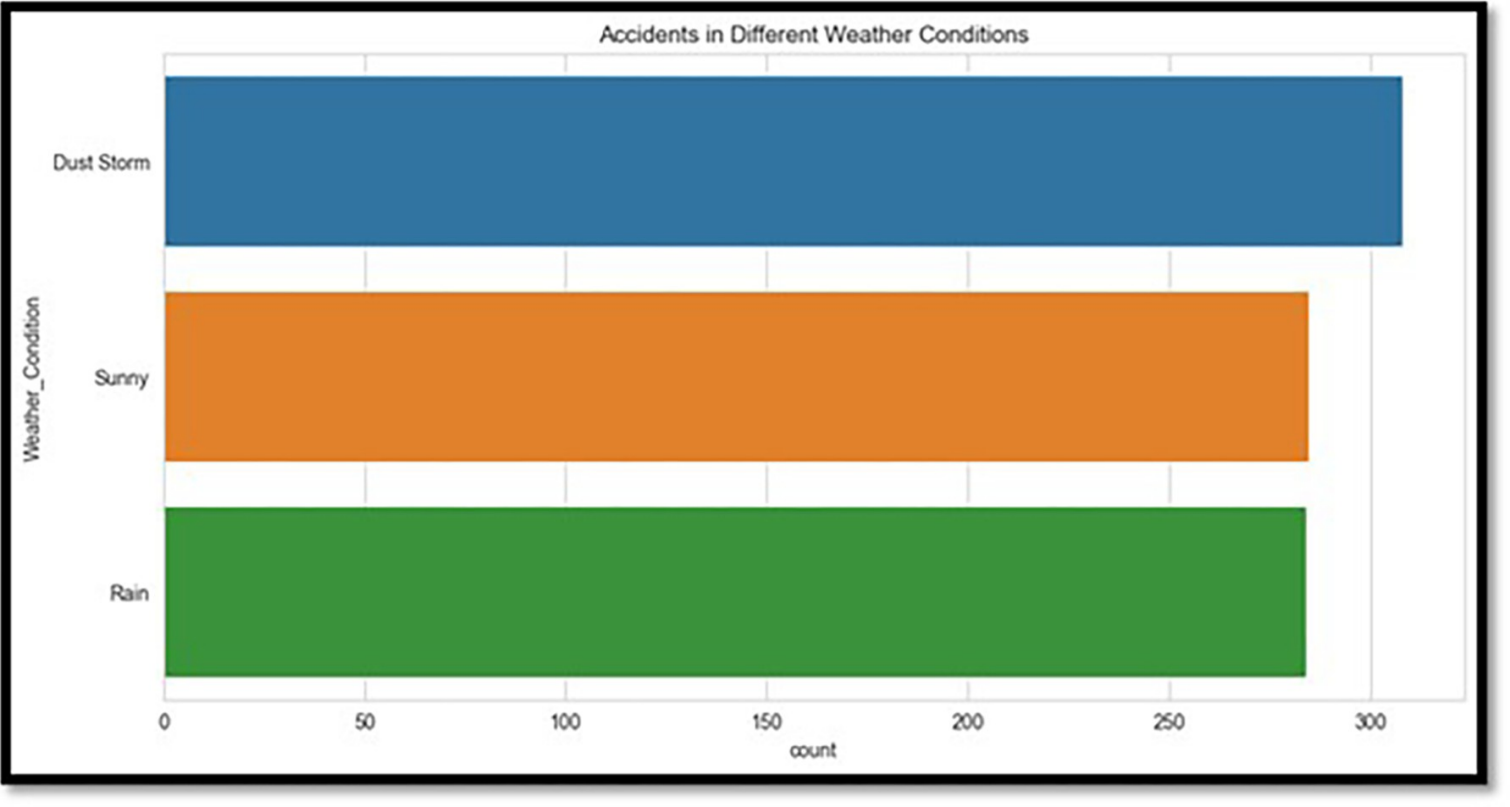
Accidents in different weather conditions.

**Fig 4:**
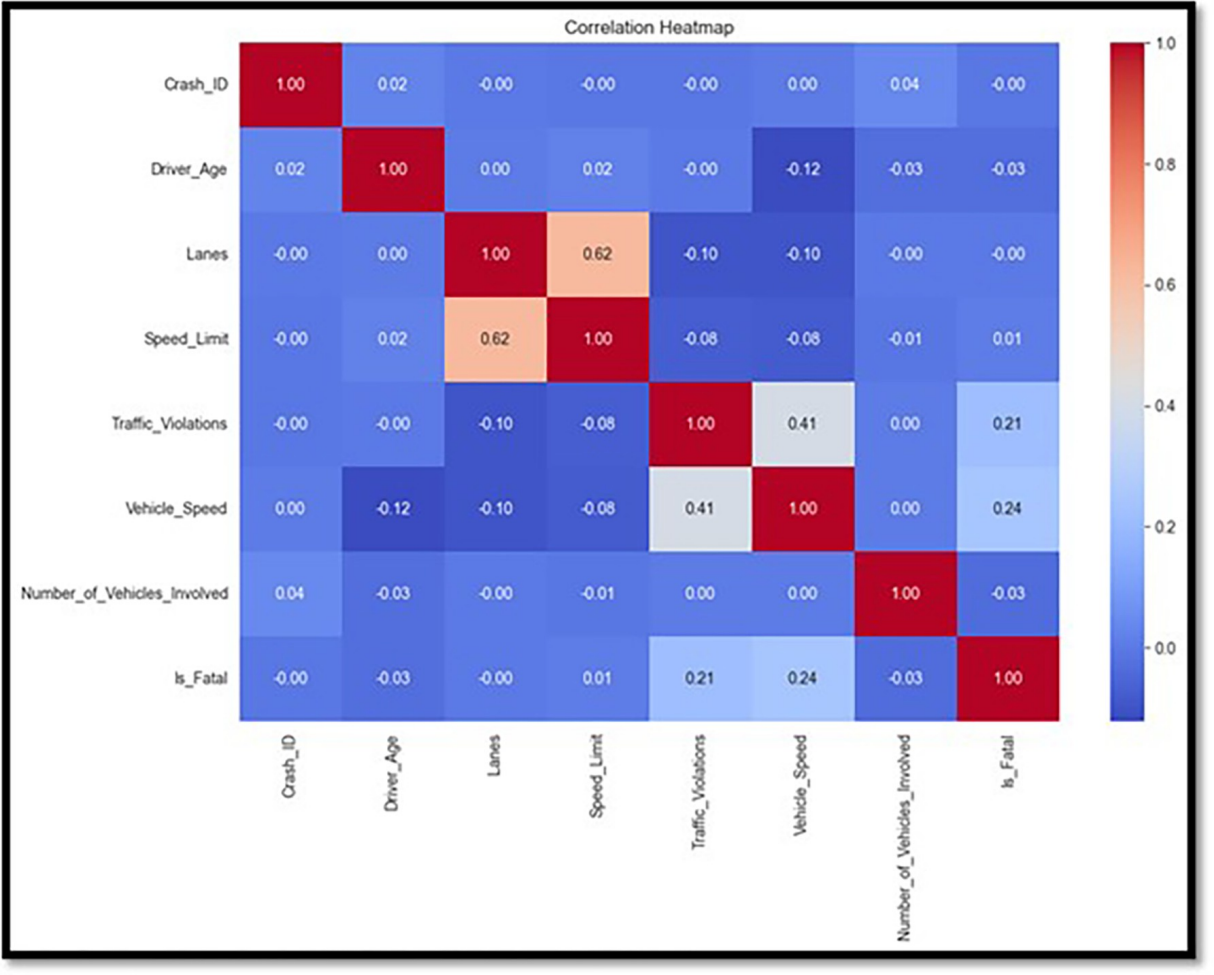
Correlation heatmap between variable.

Advanced visualization techniques were used with Python’s Matplotlib and Seaborn modules to ensure a clear and meaningful depiction of the data. The age distribution of drivers, as shown in Fig 1, is dominated by individuals in the 20–50-year age range, according to the degree of injuries. The type of crashes is shown in Fig 2, with rear-end collisions being the most frequent. Fig 3 shows accidents by weather conditions. Using a correlation heatmap, Fig 4 illustrates positive connections between fatal risk factors such as higher speed limits and older driver age. These visualizations are essential to create the foundation for more feature engineering and analysis.

### 4 Methodology

**Fig 5:**
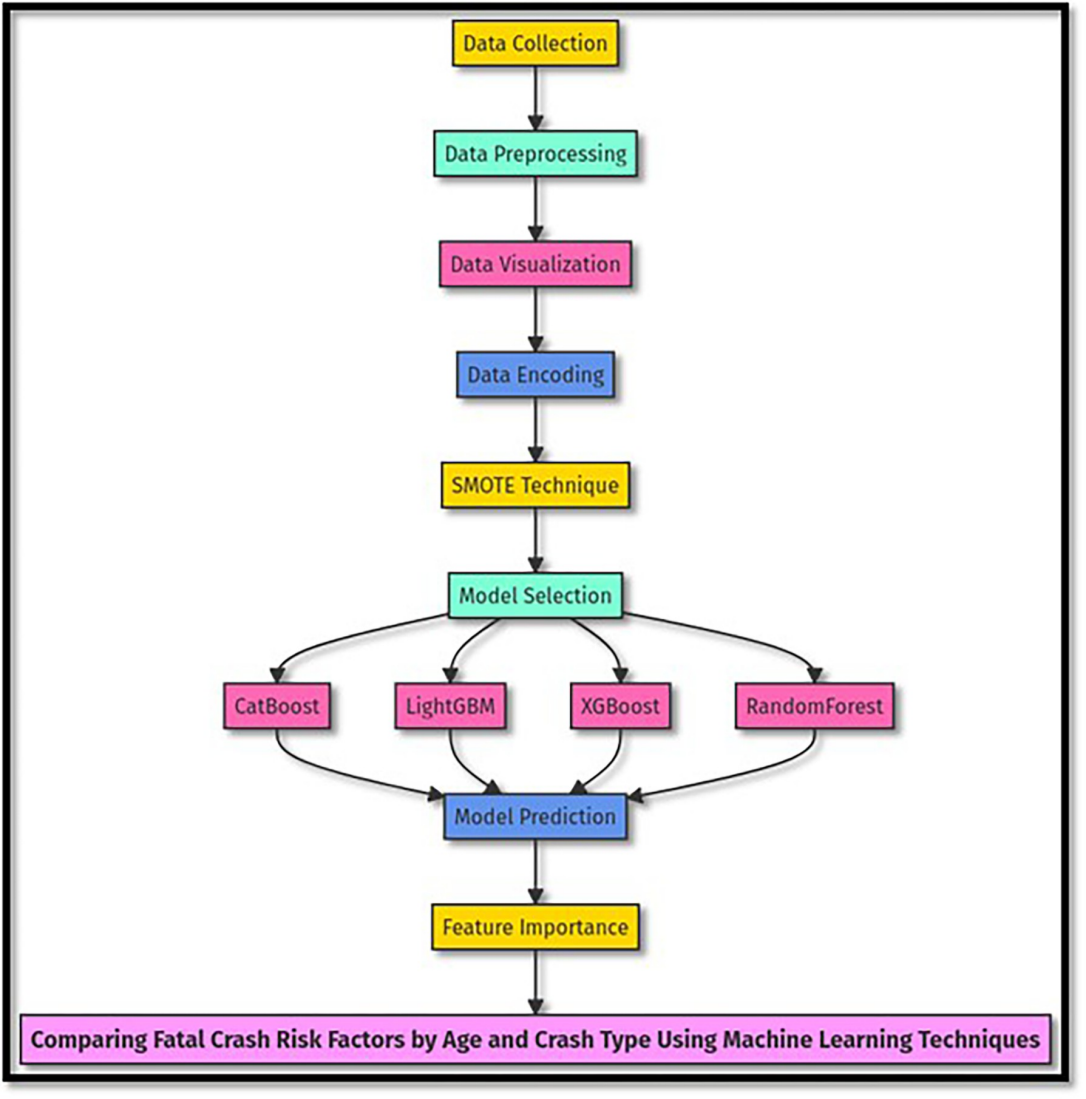
Methodology flow chart.

This study employs a comprehensive approach utilizing machine learning techniques to analyze traffic crash data from Jeddah City. The primary objective is to identify and compare risk factors associated with traffic crashes, focusing on different age groups and types of incidents. The methodology is structured as shown on Fig 5.

#### 4.1.1 Data collection and preprocessing

The dataset comprises 877 traffic accidents recorded by the Traffic Police Department of the city between 2019 and 2023. The data includes 29 variables, such as crash type, driver age, weather conditions, and severity of injuries. Preprocessing steps include one-hot encoding for categorical variables and the application of the Synthetic Minority Oversampling Technique (SMOTE) to address class imbalance.

#### 4.1.2 Model development

Four advanced machine-learning algorithms are employed: LightGBM, XGBoost, CatBoost, and Random Forest. These models are selected for their proven effectiveness in modeling complex patterns in accident data. A random search for hyperparameters is conducted on training samples to fine-tune the models for optimal accuracy.

#### 4.1.3 Performance evaluation

The models are evaluated based on accuracy, precision, recall, and F1 score. The SHAP (SHapley Additive exPlanations) values and plot dependency are also analyzed to identify significant risk factors contributing to fatal collisions.

#### 4.1.4 Feature importance analysis

The importance of various features in predicting crash severity is assessed using the LightGBM classifier. Weather conditions, time of day, and speed-related parameters are evaluated for their impact on fatal crash predictions.

#### 4.1.5 Model comparison

The performance of the machine learning models is compared to identify the most effective approach for predicting crash severity in the city. The LightGBM model demonstrates the highest accuracy, with XGBoost and CatBoost also showing competitive performance.

The methodology employed in this study showcases the novelty and contributions of our work by applying machine learning techniques to a comprehensive dataset of traffic incidents. By utilizing advanced algorithms and comparative analysis, we aim to provide a deeper understanding of the risk factors associated with traffic crashes, contributing to developing targeted safety measures and interventions.

### 4.2 Major contributions

This study makes several significant contributions to the field of traffic safety research and policy development:

#### 4.2.1 Methodological innovation

By employing a range of advanced machine learning techniques, including LightGBM, XGBoost, CatBoost, and Random Forest, this study introduces a novel approach to analyzing traffic crash data. These algorithms, coupled with a comprehensive hyperparameter tuning process, ensure the predictive models’ robustness and accuracy.

#### 4.2.2 Analysis scope

The research extends beyond traditional statistical methods by comparing risk factors associated with traffic crashes across different age groups and types of incidents. This approach offers a more nuanced understanding of the underlying causes of traffic accidents, enabling the identification of specific risk factors for targeted age groups and scenarios.

#### 4.2.3 Implications for traffic safety research and policy

The findings of this study have practical implications for traffic safety interventions and policy formulation. By highlighting key risk factors and their relative importance, policymakers and traffic safety professionals can develop more effective strategies to reduce the incidence and severity of traffic crashes. Additionally, the insights gained from this research can inform the design of customized safety programs and regulations tailored to the unique characteristics of different driver groups and road conditions.

#### 4.2.4 Contribution to academic discourse

This research contributes to the academic discourse on traffic safety by comprehensively analyzing crash risk factors using machine learning methods. The study’s methodology and findings add to the body of knowledge in the field, offering a basis for future research and exploration.

## 5 Results

The performance of all four constructed machine learning models—LightGBM, XGBoost, CatBoost, and Random Forest—on the typical 30% blind test set is compared in this portion of the paper. In addition, SHAP values and feature significance studies are used to ascertain the primary risk variables that significantly contribute to fatal collisions.

The LightGBM classifier achieved the most remarkable test accuracy of 94.9%, with XGBoost coming in a close second with a test accuracy of 95.4%. In comparison to decision trees that are used on their own, boosting-based models have been shown to have a much higher capacity for prediction. All models could match the training data correctly, achieving one hundred percent scores for accuracy, precision, and recall, suggesting no underfitting. LightGBM, on the other hand, displayed the best generalization with almost no insignificant overfitting. A score of 0.949 on the F1 test indicates a substantial equilibrium between the accuracy and recall measures. The confusion matrices that visually validate this trend are provided. These figures cover all four models. Methods that boost results in a reduction in the total number of misclassifications.

It is possible to credit LightGBM’s persistent domination to its sophisticated approaches, including leaf-wise tree growth, histogram-based data binning, and gradient-based one-side sampling, all resulting in quicker and more accurate ensembles. Regarding delivering competitive performance, XGBoost uses weighted quantile sketching to provide approximate tree splitting, while CatBoost uses ordered boosting and categorical optimization. However, it is understandable that the non-ensemble Random Forest model deviates in terms of its predictive power.

### 5.1 LightGBM model

**Fig 6:**
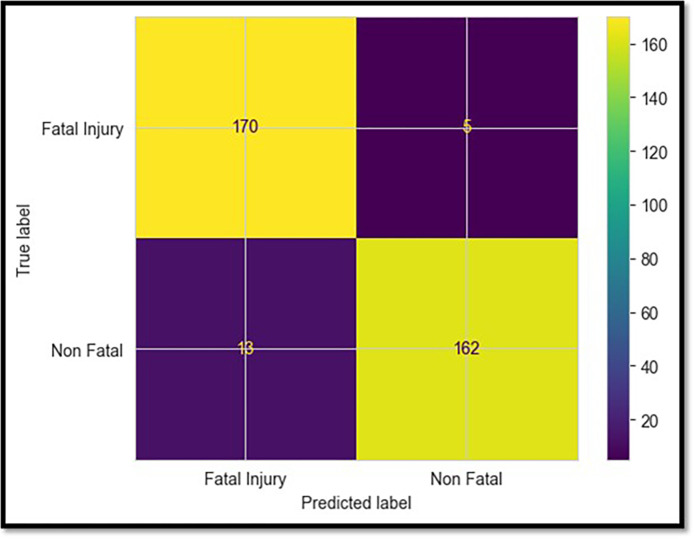
Confusion matrix for LightGBM model.

Compared to all other classifiers, the LightGBM model had the highest test performance on the essential metrics shown in Table 2. It achieved a test accuracy of 94.9% during the testing process, indicating that it accurately identified both fatal and non-fatal collisions. This suggests that there is a balanced trade-off between precision (95%) and recall (94.9%), as shown by the test F1 score of 0.949 [29]. There were incredibly few incorrect classifications, further confirmed by the confusion matrix displayed in Fig 6. The model accurately predicted 166 of the actual fatal accidents.

**Table 2:** Performance comparison of machine learning models.

Model	Algorithm Type	Accuracy (Test Set)	Precision	Recall	F1 Score
LightGBM	Gradient Boosting	94.9%	95.0%	94.9%	94.9%
XGBoost	Gradient Boosting	95.4%	95.6%	95.4%	95.4%
CatBoost	Gradient Boosting	94.0%	94.1%	94.0%	94.0%
RandomForest	Ensemble	89.1%	89.7%	89.1%	89.1%

In contrast, nine of the non-fatal instances were incorrectly classified as fatal. The total number of fatal accidents was 175. The LightGBM model showed a strong generalization capacity when identifying fatal accidents on data that had not yet been observed [30].

### 5.2 XGBoost model

**Fig 7:**
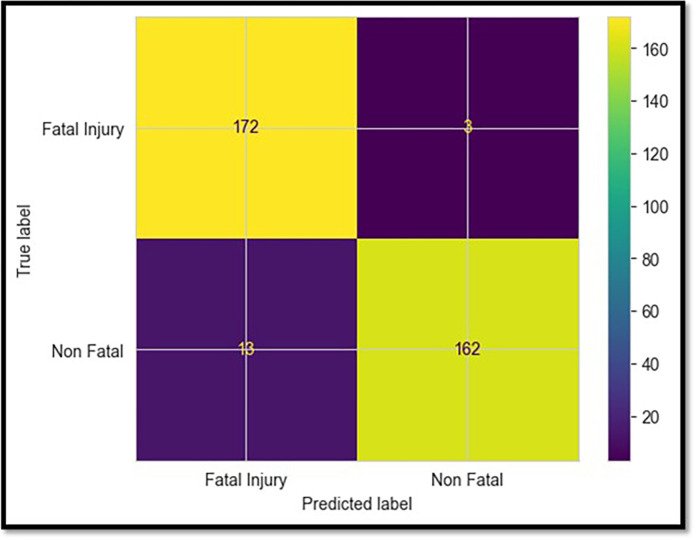
Confusion matrix for the XGBoost model.

Based on the data shown in Table 2, the XGBoost classifier achieved the second-highest test accuracy, 95.4%, behind the LightGBM classifier. In addition, its precision, recall, and F1 metrics were highly competitive, demonstrating that it completely understood the differences between deadly and non-fatal patterns. Fig 7 displays the confusion matrix, which shows that only eight fatal collisions were misjudged, compared to 167 correct predictions based on the test data. XGBoost can avoid overfitting using sophisticated optimization and regularisation methods such as weighted quantile sketching [31, 32]. This helps to strengthen the generalizability of the neural network.

### 5.3 CatBoost model

**Fig 8:**
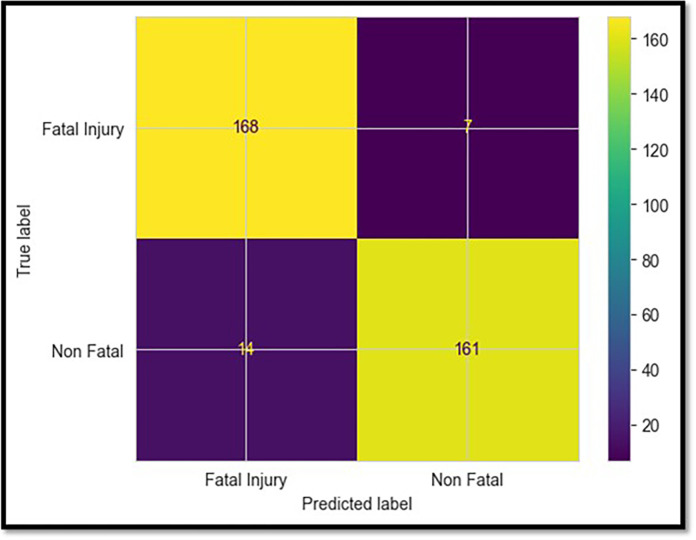
Confusion matrix for CatBoost model.

Even though it was somewhat less accurate than earlier models, the CatBoost classifier nevertheless reached a remarkable test accuracy of 94% when it came to classifying the severity levels of crashes, as seen in Table 2. Because of its high accuracy and recall level, it could differentiate between the distinct data features that apply to fatal and non-fatal collisions. Fig 8 illustrates the confusion matrix with 11 incorrect predictions out of 175 reported fatalities. Regarding tree building, CatBoost uses ordered boosting and can naturally handle categorical factors, resulting in improved performance [33, 34].

### 5.4 Random forest model

**Fig 9:**
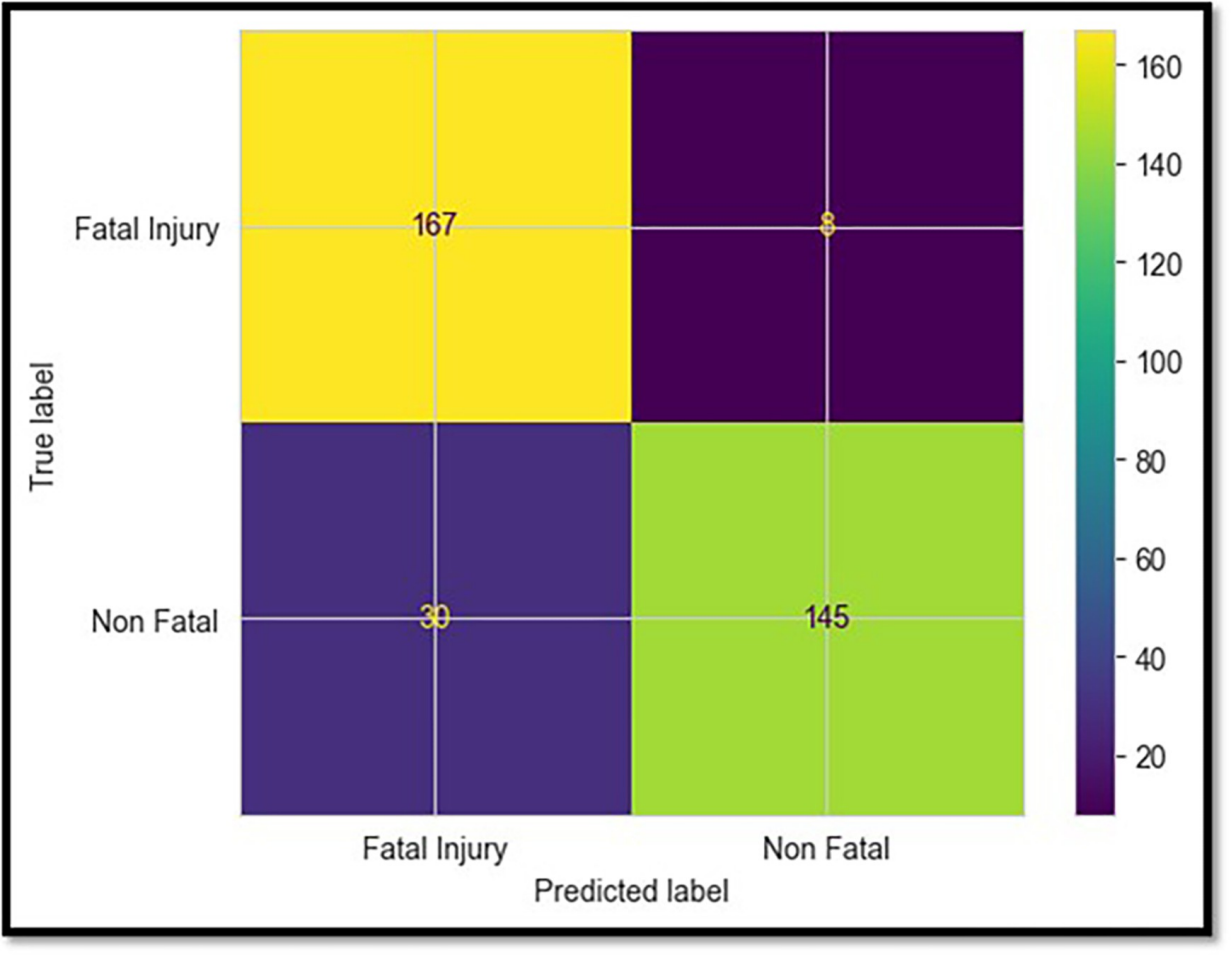
Confusion matrix of random forest model.

It should be no surprise that Random Forest, a non-ensemble model, had the lowest accuracy out of the four classifiers, with a test performance of 89.1%, according to Table 2. It also had the lowest score on the F1 scale. Consequently, this was represented in the considerably more significant misclassifications apparent in the confusion matrix shown in Fig 9. On the other hand, Random Forests provide additional benefits for crash investigation because of their interpretability and minimum tuning requirements [35, 36].

### 5.5 Performance comparison of machine learning models

The research uses accident data to evaluate four machine-learning models: LightGBM, XGBoost, CatBoost, and RandomForestClassifier. When applied to the test set, the LightGBM model demonstrates high accuracy (94.9%) and an F1 score (94.9%). At 95.4% accuracy and an F1 score, the XGBoost model performs somewhat better than the LightGBM model. The accuracy of the CatBoost model is 94%, and it has a score of 94% on the F1 scale. A lesser accuracy of 89.1% is achieved by the RandomForestClassifier, which also achieves a comparable F1 score. Each model demonstrates impressive performance, with XGBoost coming out on top somewhat in terms of accuracy and F1 score.

### 5.6 Features importance analysis

LightBGM Classifiers with the city’s Crash Data have provided insights into crash circumstances. These classifiers could distinguish acute and chronic complications and motor-based and other injuries. Weather conditions (17.8%), time of the day (13.1%), and speed-related parameters (12.5%) are factors that can influence fatality predictions. An excellent technique for interpreting complex machine learning models is offered by the LightGBM connected to SHAP analysis. This approach stresses the impact of time of the day, weather conditions, and speeding factors on the severity of crashes.

**Fig 10:**
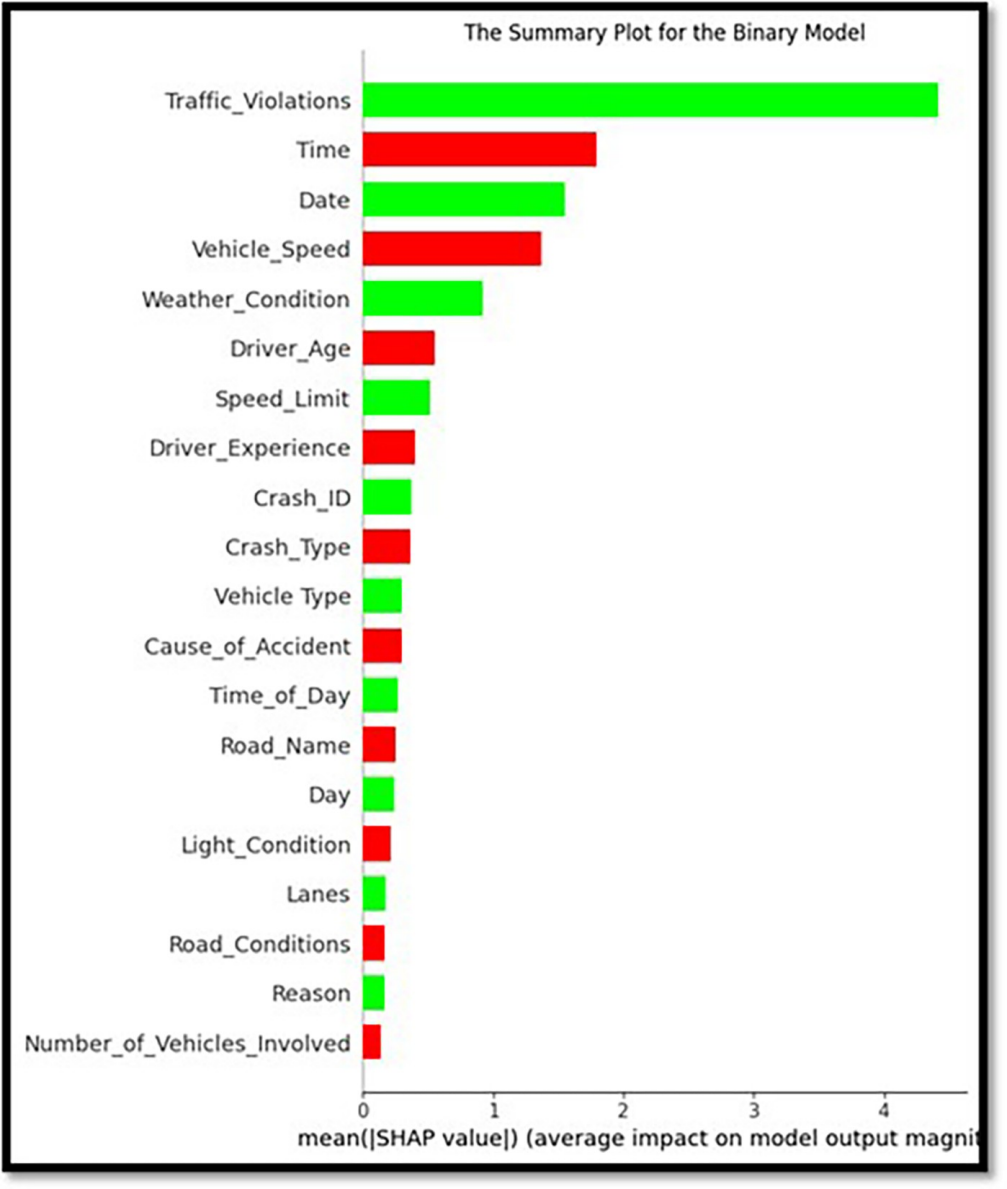
Summary plot for the binary model.

Nevertheless, an assessment of road surface characteristics and a comparison between fatal and non-fatal accidents will be conducted. To identify the effect of different factors on a fatal compared to a nonfatal outcome, SHAP value plots are used for non-fatal outcomes in a collision severity study. This proves the importance of road surface, climatic conditions, and driving attitudes, including Overspeeding. To achieve the Vision 2030 goals on road safety, there is a need for strict enforcement of traffic rules, proper maintenance of good roads, and reduced speed. However, this report highlighted the importance of features and what made Shap’s study possible for every attribute impacting the model prediction. To get a good idea of these links, the following charts (Fig 10) describe the complicated links between different factors affecting the model prediction.

**Fig 11:**
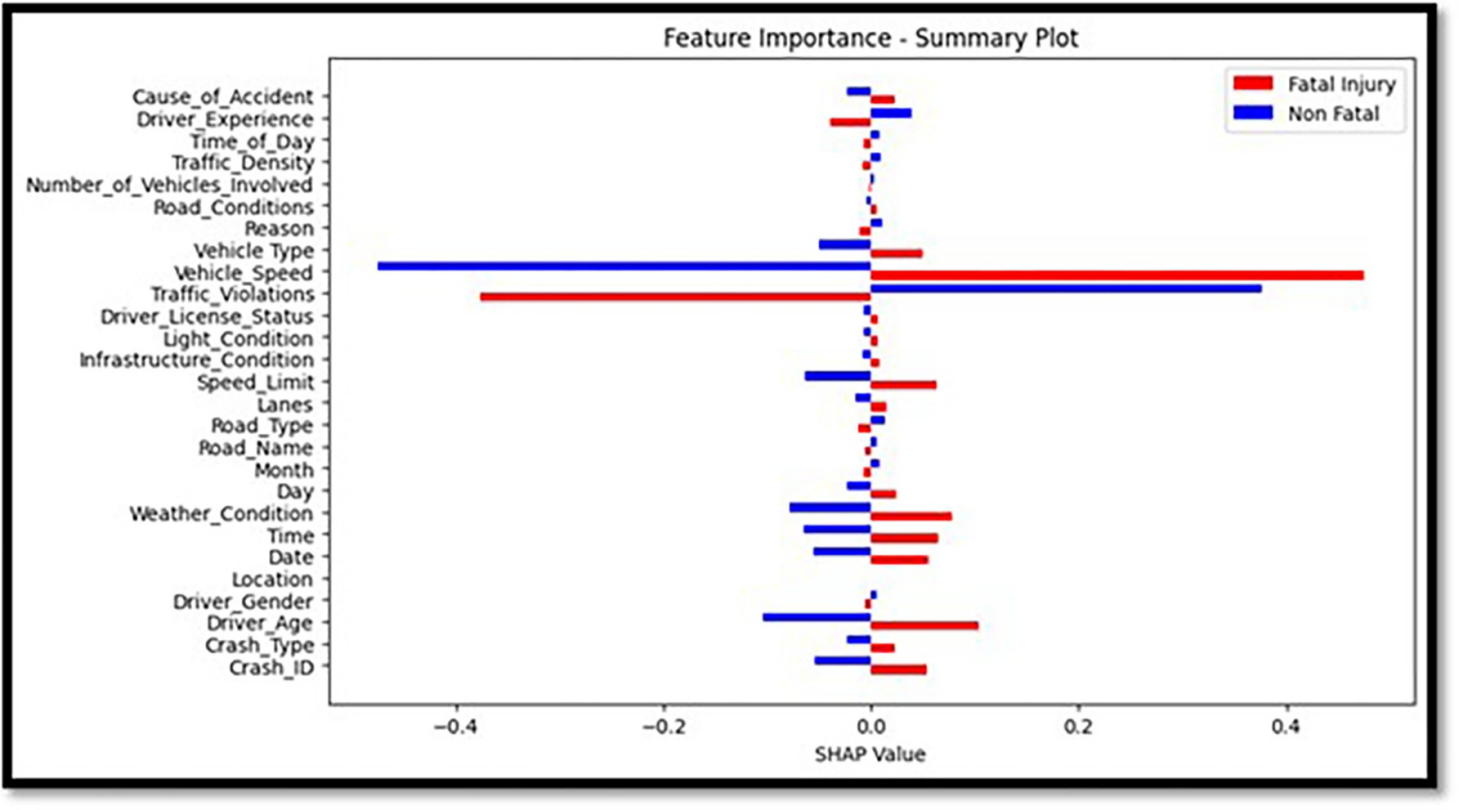
Feature importance summary plot.

The above plot depicts the binary classifier that differentiates ‘Fatal Injury’ versus ‘Non-Fatal.’ It uses a bar chart in which red and blue indicate the traits that result in categorizing each feature. This helps to make SHAP values quantify how each element affects the model’s output, and it also helps to provide a summary picture to be used for presenting feature importance in the model as shown in Fig 11.

**Fig 12:**
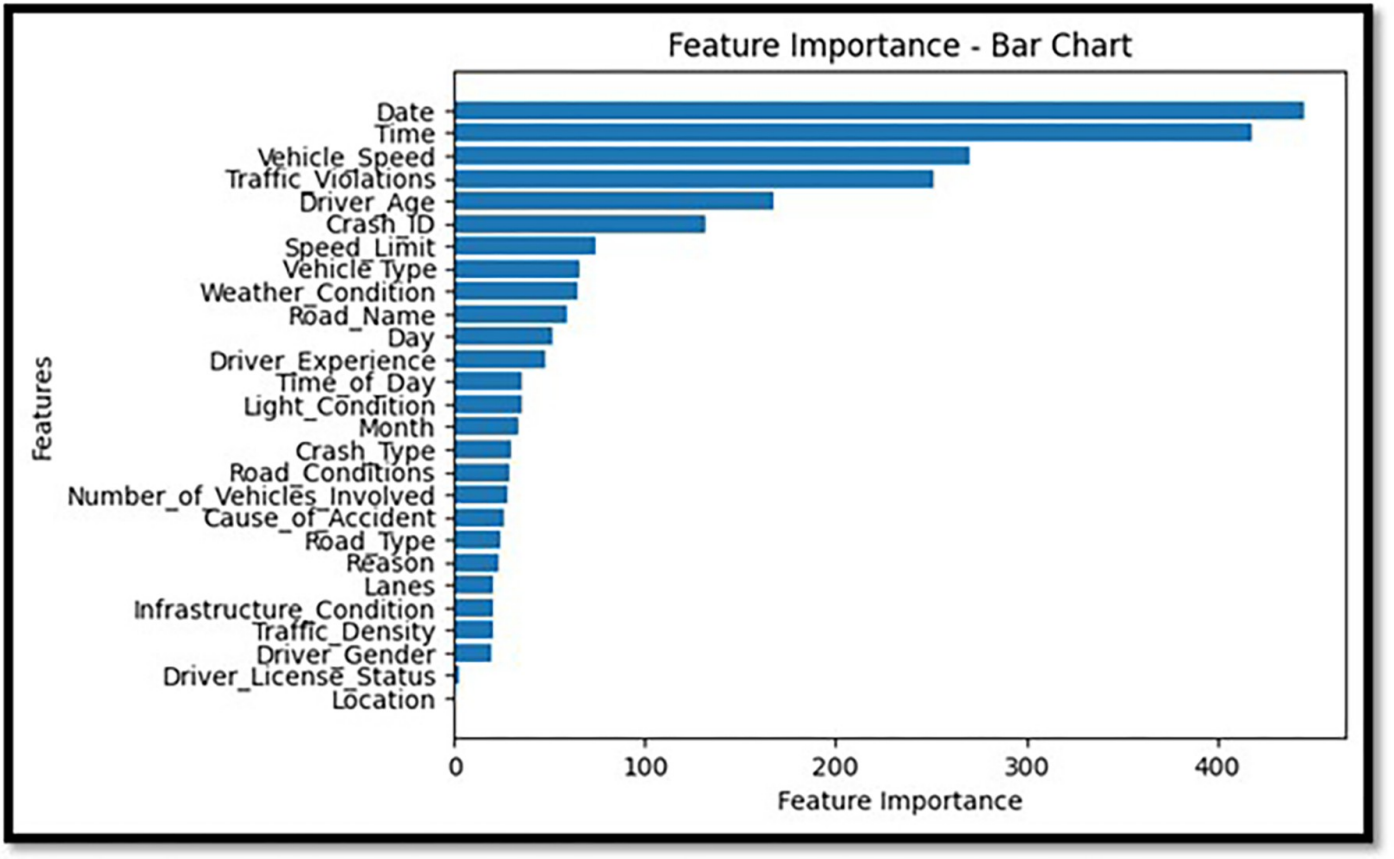
Feature importance bar chart.

The summarized map in Fig 11 is enhanced by stacking the feature relevance to fatal injuries over the feature’s relevance to non-lethal injuries. It outlines the roles that individual characteristics have in each category. A horizontal bar chart format makes it easier to compare and shows the traits that affect the most regarding collision severity. Fig 12 depicts a comprehensive image of every feature’s importance according to the LightGBM classifier. This procedure rates every feature based on its significance, with the x-axis denoting the importance level for each feature and the y-axis representing that particular feature being listed. The sorted bar chart can allow one to intuitively know which features are most influential for forecasting crash results.

**Fig 13:**
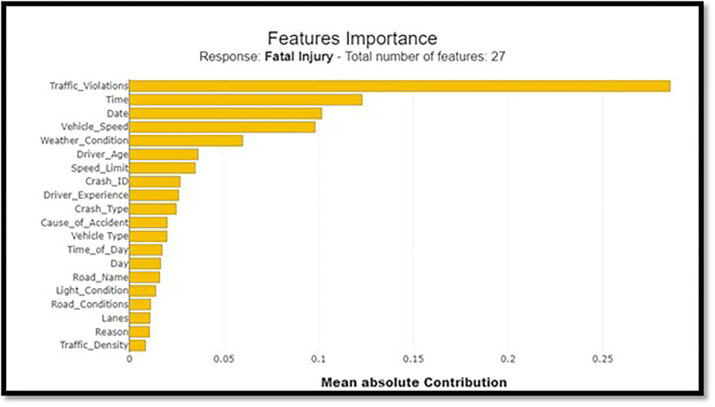
SHAP value for fatal injuries.

The SHAP values for fatal injuries are shown in Fig 13. This figure derived from the SHAP analysis was designed to concentrate mainly on the ’Fatal Injury’ categorization. It illustrates each attribute’s average influence on the model’s performance in predicting fatal events. In addition to adding a layer of interpretability, the use of SHAP values reveals the direction and size of the effect exerted by each characteristic.

**Fig 14:**
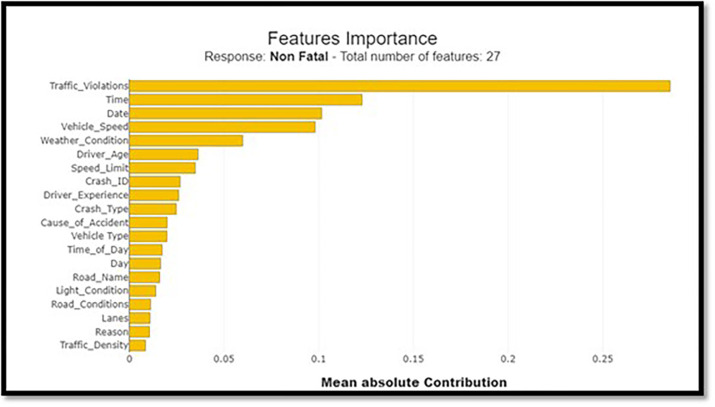
SHAP value for non-fatal injuries.

Fig 14 displays the SHAP values for non-fatal outcomes. This chart continues Fig 4, focusing on the ’non-fatal’ categorization. It illustrates how each characteristic influences the model’s predictions, resulting in less deadly outcomes. This graphic is essential to thoroughly understand the diverse impacts of features in less severe crash situations.

## 6 Discussion

### 6.1 Analyzing predictive features

Our machine learning algorithms identified several critical characteristics highly predictive of the likelihood of fatal collisions. The most relevant factors were the driver’s age and gender, weather conditions, time of day, speed-related variables, and road conditions. These findings are consistent with theoretical understandings of how driving behavior and various circumstances affect accident severity. For example, younger male drivers tend to engage in riskier driving habits, and reduced visibility during adverse weather conditions leads to slower response times. Similarly, high driving speeds also impact vehicular control.

### 6.2 Practical implications

Speeding characteristics have a considerable predictive effect, necessitating targeted speed enforcement and traffic calming measures. Installing speed cameras, modifying speed limits according to road construction, and imposing stricter fines for speed limit violations are potential solutions to address this widespread risk factor. Additionally, the dangers associated with younger male drivers highlight deficiencies in graduated driver licensing systems and underscore the need for defensive driving education campaigns targeting high-risk demographic groups. Given the potential impact of adverse weather conditions, transportation authorities may consider increasing the use of variable speed limit systems or advisory alerts triggered by unfavorable conditions.

### 6.3 Comparison with past literature

Our research lends credence to previously held beliefs on the primary factors determining collision severity. Previous studies at a macro level have consistently found characteristics such as speeding, driver age, gender, and adverse weather as significant risk factors in both developed and developing nation settings. Our findings provide further evidence that human behavior, infrastructure design, and environmental factors all play an essential part in determining the outcomes of various collisions. Incorporating new predictive variables, such as the time of day, enhances explanatory power and reveals other situational elements influencing collision likelihood. Our micro-level research of an understudied setting provides additional detail on localized accident trends.

### 6.4 Methodological reflections

The use of ensemble machine learning approaches such as XGBoost, which offer predictive advantages over traditional statistical models due to their ability to capture complex interacting effects, was a significant strength of this work. However, the model inputs were limited, as police reports were the sole data source. Incorporating hospital trauma records could provide a deeper understanding of injury patterns and accident consequences. More comprehensive traffic volume measurements could also help isolate exposure concerns across various scenarios. Deep learning approaches might further assist models in inferring non-linear predictive relationships within such multidimensional data.

### 6.5 Limitations and future research

While providing valuable insights into traffic crash risk factors in the city, this study has limitations. Firstly, the dataset used in this research was limited to recorded traffic incidents by the Traffic Police Department, which may not capture all relevant variables or the extent of traffic crashes in the area. Additionally, the reliance on police reports may introduce bias, as not all incidents are reported or recorded accurately.

Methodologically, the study employed machine learning techniques that, while effective, may not fully account for the complexity of traffic safety dynamics. The models used depend on the quality and completeness of the input data, and the results may vary with different datasets or contexts.

The applicability of the study’s findings is primarily limited to the urban setting of the city. The risk factors identified and the model’s predictions may not be directly transferable to other regions or countries with different traffic conditions, cultural factors, or road safety policies.

Future research should address these limitations by expanding the dataset to include a broader range of variables and incidents. Incorporating data from other sources, such as hospital records or traffic surveillance systems, could provide a more comprehensive understanding of crash severity and its determinants. Exploring other machine learning techniques or deep learning models may offer further insights into the complex interactions between risk factors.

Further studies could also focus on the generalizability of the findings, testing the models in different geographical settings or applying the methodology to other types of traffic safety issues. Developing more tailored intervention strategies based on the specific risk factors identified in this study could significantly contribute to reducing traffic-related injuries and fatalities, both in Saudi Arabia and globally.

This section should be placed at the end of your paper, following the Discussion section. Please let me know if you need any further assistance or modifications.

### 6.6 Broader societal impacts

Our models map out potential avenues for targeted safety interventions that contribute to achieving Saudi Vision 2030 quality-of-life goals. These initiatives aim to reduce the hazards among younger drivers and other categories. Realizing further prediction improvements via data integration might put Saudi transportation authorities in a better position to undertake risk-proportionate countermeasures, optimize resource allocation, and achieve sustained reductions in traffic casualties. In a broader sense, predictive analytics provide options not before recognized for evidence-driven traffic safety planning in emerging nations that are fast becoming more motorized.

## 7 Conclusion

This research aimed to give unique insights into differential fatal accident risk variables by using machine learning methodology approaches. As a result of analyzing more than 800 incidents using models such as LightGBM, XGBoost, CatBoost, and Random Forest, important conclusions were discovered that have substantial implications for traffic safety.

- The models made A successful distinction between fatal and non-fatal outcomes, with LightGBM displaying the most remarkable accuracy of 94.9%. Some significant risk factors, including unfavorable weather conditions, driving late at night or early in the morning, and speeding characteristics, were shown to be among the most significant predictors of fatal crashes using characteristic significance analyses.
- The findings of this study demonstrate how environmental, temporal, and behavioral dynamics significantly impact the severity of the consequences of collisions.
- These results indicate increased dangers in some conditions and among specific drivers, which should be considered when formulating targeted legislation, practices, and safer road campaigns.
- Some approaches, such as adaptive speed control, good road management during rainy seasons, and age-specific safety initiatives, if added to the existing plan being developed in line with Vision 203,0, could go a long way in mitigating these deaths and injury incidences.
- This landmark study demonstrates the utility of machine learning in comprehending complex causes of a crash factor even though certain obstacles involve more crash characteristics and inter-national factors, which should suggest opportunities for follow-up research.
- There has to be continuity of research in this category as it is crucial for a complete resolution of global road safety problems through personalized information-based measures that reflect various risks. This research contributes significantly towards this goal and opens other directions that might imply additional applications with significant consequences.
